# Human IgE responses to different splice variants of *Schistosoma mansoni* tropomyosin: associations with immunity^☆^

**DOI:** 10.1016/j.ijpara.2014.02.004

**Published:** 2014-05

**Authors:** Sukrit Silas, Colin M. Fitzsimmons, Frances M. Jones, Angela Pinot de Moira, Jakub Wawrzyniak, Edridah M. Tukahebwa, David W. Dunne

**Affiliations:** a University of Cambridge, Department of Pathology, Tennis Court Road, Cambridge CB21QP, UK; b Vector Control Division, Ugandan Ministry of Health, Kampala, Uganda

**Keywords:** *Schistosoma mansoni*, Tropomyosin, Alternative splicing, Human IgE

## Abstract

Resistance to *Schistosoma mansoni* infection has been correlated with IgE responses to the adult worm. Molecular targets of this response are gaining interest as markers of immunity and as indicators of allergenic properties. Few protein families contain IgE antigens (allergens) and one of the most highly represented are the tropomyosins. Alternative splicing generates numerous tropomyosin isoforms, which in parasites is likely to induce a range of anti-tropomyosin responses in the host. Here we examine human IgE and the counteracting IgG4 responses to splice variants of *S. mansoni* tropomyosin (SmTpm)*.* It was possible to show life-cycle transcription profiles for 12 of 20 predicted splice variants from the four SmTpm genes. We expressed recombinant protein of four variants of TpmII (TpmII.4, 8, 3 and 7) with considerable differences in sequence. TpmII.4 and 8 were muscle, and TpmII.3 and 7 non-muscle, types. IgE and IgG4 responses to all four proteins were measured in a population of 228 infected boys and men (7–76 years) from a region of Uganda endemic for *S. mansoni*. Levels of these antibodies were not dependent on age and did not change following anthelminthic treatment. IgE to TpmII.3 was common in the cohort (>60%) and IgG4 to TpmII.3 less so (33%). IgE to TpmII.7 was rare (6.5%), but IgG4 to TpmII.7 was more common (49%). In regression analysis, a detectable IgE response to TpmII.3 was associated with reduced re-infection 2 years after treatment and an IgG4 response to TpmII.7 with increased re-infection. Different isoforms generated by alternative splicing are targeted by different components of the anti-Tpm IgE/IgG4 response. Only some of these are associated with immunity.

## Highlights

• Tropomyosin (Tpm) is a common IgE antigen in invertebrates.
• Alternative splicing generates at least 13 Tpm isoforms in *Schistosoma mansoni*.
• Four different isoforms of *S. mansoni* TpmII (SmTpmII.3, 4, 7 and 8) were expressed.
• IgE and IgG4 responses to isoforms were measured in 228 *S. mansoni*-infected males.
• IgE to SmTpmII.3 was associated with reduced re-infection 2 years after treatment.

## Graphical abstract

**Figure:**
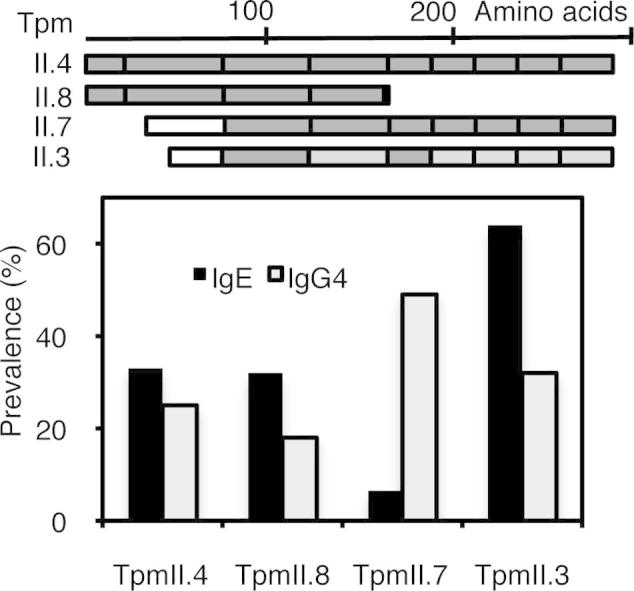

## 1 Introduction

Infection with *Schistosoma mansoni* is a serious health problem in sub-Saharan Africa. For people living where the parasite is endemic, repeated exposure to infectious cercariae can result in life-long infection. Partial immunity does gradually develop and epidemiological studies show that this is correlated with an IgE response to the worm (Rihet et al., 1991; Dunne et al., 1992, 1997; Pinot de Moira et al., 2010; Fitzsimmons et al., 2012b). In fact, parasite-specific IgE has been associated with protection in a wide range of helminth infections (Hagan et al., 1991; Dunne et al., 1992; Faulkner et al., 2002; Bethony et al., 2005; Capron et al., 2005; Turner et al., 2005) leading to the widely-held belief that IgE evolved to help counter these multicellular parasites.

IgE also plays a central role in allergy and some propose that allergic responses are anti-parasite responses misdirected to otherwise harmless species (Artis et al., 2012). We have suggested that not only are the IgE-mediated processes in allergic and anti-worm responses the same, but so are the types of molecules targeted (Fitzsimmons and Dunne, 2009). Allergens come from a small number of protein families (Radauer et al., 2008) and the anti-worm IgE targets discovered thus far are all from these families (Fitzsimmons and Dunne, 2009). For example, the EF-hand proteins are some of most common molecular allergens (Radauer et al., 2008) and in *S. mansoni,* the EF-hand-containing Tegumental-allergen-like (SmTAL) protein family are dominant IgE antigens. IgE responses to a number of these proteins (SmTAL1, 3 and 5) have been associated with resistance to infection (Dunne et al., 1997; Fitzsimmons et al., 2012b).

Schistosome tropomyosin (Tpm) is another likely target for the host IgE response. Tpm has been described as an invertebrate “panallergen” (Reese et al., 1999) with over 45 allergenic examples in species ranging from shellfish and dust mites to the parasitic nematode *Anisakis simplex* (Asturias et al., 2000; Resch et al., 2011). The canonical view is that invertebrate Tpms sharing <55% identity with their human homologues may cause allergy whereas more similar (e.g. mammalian) Tpms do not (Jenkins et al., 2007). Within any species there can be as many as 40 different Tpm isoforms. These serve a range of functions in different tissues (Schevzov et al., 2005), at different developmental stages and in both muscle contraction and cytoskeletal structure/function (Perry, 2001; Gunning et al., 2005). In vertebrates the multiple isoforms are achieved through extensive differential RNA splicing (Perry, 2001) and there is evidence that this is also the case for invertebrates (Jeong et al., 2004; Alvite and Esteves, 2009). IgE binding has been examined for a number of allergenic Tpm proteins and epitopes have been predicted (Sereda et al., 2008), but the issue of multiple isoforms has not been addressed. Different isoforms can vary considerably in regions of the molecule, and this is likely to affect their antigenicity (Jeong et al., 2004).

Alternative splicing of *S. mansoni* Tpm (SmTpm) is predicted from genomic studies (Berriman et al., 2009) but it is not known whether different isoforms are expressed in different parasite tissues or in different life stages. There is evidence from other allergen-like molecules that timing and location of expression are important in the natural history of the infection and in the host immune response (Fitzsimmons et al., 2012a). In this report we examine the life cycle transcription of splice variants of the four *S. mansoni* genes confirming transcription of 12 Tpm isoforms and, focusing on one gene (SmTpmII), select four splice variants with markedly different domain structures, express these as recombinant proteins and assess IgE and IgG4 responses to the four molecules in a cohort of infected males from a region endemic for *S. mansoni*.

## 2 Materials and methods

### 2.1 Parasite cDNA preparation

Total RNA from *S. mansoni* eggs, cercariae, cercarial heads, cercarial tails, 24 h schistosomulae and 7 week adults were prepared as previously described (Chalmers et al., 2008; Fitzsimmons et al., 2012a). Total RNA from miracidia, 24 h sporocysts, and 99 h sporocysts was a gift from Prof. T. Yoshino (University of Wisconsin, USA). All parasite RNA samples were treated with DNAse I (Roche, Germany) to remove genomic DNA contamination and RNA integrity was assayed using an Agilent 2100 BioAnalyzer. cDNA was synthesised from 1 μg of total RNA from each life cycle stage using SuperScript II Reverse Transcriptase (Invitrogen, Life Technologies, UK) and random primers, and verified to be free of parasite genomic DNA contamination by PCR using intron-flanking primers 5′-AAC TTT ACT TCG TGA TGG TGA TGA-3′ and 5′-ACC ACG ACA AAA TTC TTC AAA AGT-3′ from constitutively expressed SmTAL2 (Fitzsimmons et al., 2012a).

### 2.2 Detection of Tpm isoforms

Genes annotated as Tpms in the *S. mansoni* genome (Berriman et al., 2009) on GeneDB (Sanger Institute, http://www.genedb.org/Homepage/Smansoni). Smp_022170, Smp_031770, Smp_044010, Smp_085290 and Smp_032490 were analysed for conserved domains by BLAST searches of NCBI/GenBank databases (https://www.ncbi.nlm.nih.gov/genbank/) and sets of primers were designed to uniquely identify each putative alternatively spliced isoform of every gene by PCR (Supplementary Table S1). In cases where this was impossible, primer pairs were generated to carry out nested PCR (Supplementary Table S2). Wherever possible, primers were designed to span introns. All PCRs in this study were performed using Phusion Hot-Start High-Fidelity DNA Polymerase (New England Biolabs, UK).

All reactions were performed according to the following cycling protocol: an initial denaturation at 98 °C for 30 s, 35 three-step cycles of 98 °C for 10 s, *T*_anneal_ for 20 s and 72 °C for 30 s, followed by a final extension step at 72 °C for 5 min, on a DNA Engine Tetrad 2 Peltier Thermocycle (Bio-Rad, UK). The reaction products were then visualised by ethidium bromide (EtBr)/agarose gel electrophoresis. In the case of a negative result, the reaction was repeated at annealing temperatures 2–4 °C lower than the optimum, until non-specific binding of the primers became apparent.

The primers for Smp_031770.3 (TpmII.3) were later found to not be specific to this variant alone; they also amplify the novel isoform Smp_031770.16 (TpmII.16) identified in this study.

### 2.3 Cloning and sequence verification

The coding DNA sequences (CDS) of four SmTpm II (Smp_031770) variants, TpmII.3, 4, 7 and 8, were amplified from cercarial cDNA using the following primers: 5′-ATG AAG CTT CAG ATA GAC CAGC-3′ and 5′-TTA GAT ATT TTC TAC TTC AGT AAA CAT GG-3′ for TpmII.3 (699 bp), 5′-ATG GAA CAT ATT AAA AAG AAA-3′ and 5′-TTA GTT TCC AGT AAG TTC TG-3′ for TpmII.4 (855 bp), 5′-ATG GAA GAA GCT TTA TCA G-3′ and 5′-TTA GTT TCC AGT AAG TTC TG-3′ for TpmII.7 (720 bp) and 5′-ATG GAA CAT ATT AAA AAG AAA-3′ and 5′-TTA GAA ATG CAC CTC ATC-3′ for TpmII.8 (504 bp). Amplicons were gel-purified and cloned directly into the pJET1.2/blunt vector using the blunt-ended CloneJET PCR cloning kit (Fermentas, UK). Recombinant plasmids were sequenced (DNA Sequencing Facility Cambridge University, Department of Genetics, UK). The mRNA coding sequence of SmTpmII.3, 4, 7 and 8 were uploaded to GenBank (Accession Nos. KC904503.1, KC904504.1 KC904505.1 and KC904506.1). While cloning the CDS of Smp_031770.3 (TpmII.3), a novel 699 bp isoform that was not predicted by EVM2 in the *S. mansoni* genome was found. This sequence was uploaded to GenBank (KC904507.1) as Smp_031770.16 (TpmII.16).

### 2.4 Quantitative real-time PCR (qPCR)

Transcription levels of the common TpmII exon E and of control gene *S. mansoni* RNA Polymerase II (RpII) were assayed by qPCR using the DyNAmo Flash SYBR Green qPCR Kit (Fermentas). One microlitre of diluted cDNA (corresponding to 5 ng of total RNA) was used as the maximum template amount per 20 μl reaction. Primers 5′-GCT GAA GTC GCC TCA CTA CA-3′ with 5′-TTT CAT CAG CAG CTT TAC TTG C-3′ for TpmII exon E (121 bp) and 5′-CGAAAATGATGTCAGCCTCA-3′ with 5′-CCGTTCTTGTGATTCCTCGT-3′ for RPII, were used at a final concentration of 0.521 μM. Reactions were performed according to the recommended cycling protocol for the kit, on an Eco Real-Time PCR System (Illumina, UK) and high-resolution melt curves were performed over a 55–98 °C range. qPCR amplicons were cloned into pJET1.2/blunt and sequenced as described in Section 2.3.

Standard curves were performed over seven successive twofold dilutions of the cercarial cDNA template in triplicate. Quantification was then performed in quadruplicate, using cDNA from each of the life cycle stages. No-template controls were included for each primer pair and no-primer controls were included for cDNA from each life cycle stage. Data were analysed by the 2^ΔCt^ method (Livak and Schmittgen, 2001).

### 2.5 Antigen expression and purification

Full CDS for TpmII variants 3, 4, 7 and 8 were amplified by PCR from the recombinant plasmids (see Section 2.4) using specific primers with appended restriction sites (Supplementary Table S3). Amplicons were ligated into the pGEX-KG bacterial expression vector between *Bam*HI and *Xba*I restriction sites (TpmII.3, 4 and 8) or between *Xba*I and *Xho*I sites (TpmII.7). Constructs were sequenced to verify inserted sequences. GST-TpmII fusion proteins (5′ GST) were expressed in *Escherichia coli*, isolated by affinity chromatography on glutathione–agarose (GE Healthcare, UK), reapplied to glutathione–agarose in PBS containing 1% Triton X-100 (Sigma–Aldrich, UK), washed with PBS and then cleaved at 22 °C for 16 h with 50 units (U) of thrombin per mg of fusion protein to release free TpmII.3, 7 or 8 and 4 h with 30 U of thrombin per mg of fusion protein to release TpmII.4. In each case the absence of contaminating GST was confirmed by ELISA using rabbit-anti-GST antisera (Sigma) and horseradish peroxidase (HRP)-conjugated anti-rabbit IgG (Sigma).

### 2.6 Far UV circular dichroism (CD)

Secondary structure determinations were performed using an Aviv 410 CD Spectrometer (Aviv Biomedical, USA). Antigens were prepared at a concentration of 100–200 μg/ml in a 10 mM phosphate buffer, pH 7.5, containing 50 mM NaF. Thermal denaturation of the antigens was followed at 222 nm from 25 to 95 °C in 2 °C steps with an equilibration time of 30 s and an averaging time of 1 s at a 1 nm bandwidth. Renaturation was followed from 51 °C for TpmII.4, and 8, from 81 °C for TpmII.3, and from 75 °C for TpmII.7. Wavelength scans were performed before and after thermal denaturation at 25 °C between 190 and 260 nm in 0.5 nm intervals with an averaging time of 1 s at a 1 nm bandwidth. Baseline values were obtained by concurrent CD spectrometry of buffer alone. Dynode voltages did not fall outside of the linear range in any scans. Melting temperatures were calculated by taking the maxima of the derivatives of thermal denaturation curves, and percent helicity calculations were performed using the CONTINLL analysis program (van Stokkum et al., 1990) and the SP175 190–240 nm reference data set (Lees et al., 2006) on the Dichroweb interface (Whitmore and Wallace, 2008).

### 2.7 Study cohort

IgE and IgG4 responses to TpmII variants 3, 4, 7 and 8 were measured in a sample of adult males resident in the fishing village Musoli, located on Lake Victoria, Uganda. Full details of the study area, population and sample selection have been described elsewhere (Fitzsimmons et al., 2012a). Briefly, an initial demographic survey and parasitological screen was conducted on the entire population of Musoli, and 228 *S. mansoni*-infected male inhabitants aged 7–76 years were selected by simple random sampling (Fitzsimmons et al., 2012a). Written informed consent was obtained from all selected individuals ⩾15 years who agreed to participate, or from parents/guardians of selected children <15 years giving assent. Five stool samples were taken from each individual on different days and quantitative parasitology carried out (two Kato-Katz thick smear slides per sample). The median infectious burden for this cohort was 477 *S. mansoni* eggs per gram (epg) of faeces (range 3–7,083 epg). Blood samples were collected from participants before and 9 weeks after treatment with 40 mg/kg of Praziquantel (PZQ). Of the 228 selected males, 216 donated blood samples before treatment and 202 donated samples after treatment. To determine the efficacy of treatment and intensity of re-infection, quantitative parasitology was also carried out at 9 weeks, 8 months and 2 years post-treatment. In line with Ugandan national health policy, infected participants were treated at 2 years (but not at 9 weeks or 8 months).

Ethical clearance for the study was obtained from the Uganda National Council of Science and Technology (ethics committee for Vector Control Division, Ugandan Ministry of Health).

### 2.8 ELISA with human plasma

Plasma IgE and IgG4 levels were measured by isotype-specific ELISA. A coating inhibition assay was employed to estimate the saturating coating concentrations for each antigen (20, 21, 15 and 6 μg/ml for TpmII.3, 4, 7 and 8) as described (Steinitz and Baraz, 2000; Fitzsimmons et al., 2012a) and 15 μl of each antigen were dried down onto 384-well high-binding microplates (Greiner Bio-one, UK) in 0.1 M sodium carbonate /bicarbonate buffer pH 9.6 at 22 °C. The assays were then continued exactly as described previously (Fitzsimmons et al., 2012a,b). Briefly, plates were blocked with 1% non-fat dry milk powder and incubated with plasma samples diluted 1:200 (IgG4) or 1:20 (IgE). Wells were then incubated with 0.5 μg/ml of biotinylated mouse anti-human IgE (Clone G7-18, BD Pharmingen, UK), or IgG4 (Clone JDC-14, BD Pharmingen), washed and then incubated with 1:3000 streptavidin–biotinylated HRP complex (Mast Group Ltd., UK). Finally wells were washed again and developed with l *o*-phenylenediamine substrate solution (Sigma). Plasma samples collected from 26 uninfected Europeans (after written informed consent was obtained) were tested concurrently with each assay, and all measurements were performed in duplicate. O.D. values were read using a Powerwave HT microplate spectrophotometer (BioTek Instruments Inc., UK) at 490 nm. Standard curves were generated for every plate with the O.D. values obtained from the wells coated with serial dilutions of human myeloma IgE (Calbiochem, USA) or IgG4 (Sigma).

### 2.9 Regression analysis

The influence of post-treatment anti-TpmII antibody (IgG4 and IgE) levels on intensity of re-infection 2 years post-treatment was examined using linear regression analysis, with adjustment for age. Analysis was restricted to individuals for whom treatment was efficacious, defined as at least a 90% reduction in epg and <100 epg 9 weeks post-treatment. Due to positive skew, both egg counts (epg) and anti-TpmII antibody levels were log transformed after the assay cut-offs were added to remove zero values. Age was included as a categorical variable, comprising five groups of approximately equal size. Log-likelihood ratio tests were used to determine significance. *P* < 0.05 was considered significant.

## 3 Results

### 3.1 At least 12 Tpm isoforms are present at the mRNA level in *S. mansoni*

There are five genes annotated as Tpm in the *S. mansoni* genome: Smp_044010 (TpmI), Smp_031770 (TpmII), Smp_022170, Smp_085290 and Smp_032490. However, as a result of alternative splicing it is predicted that there may be as many as 20 Tpm isoforms in the organism (Berriman et al., 2009). BLAST searches of the NCBI/GenBank databases were performed to identify conserved Tpm domains in the *S*. *mansoni* genome. Smp_032490 was excluded from further analysis as it did not map onto any known Tpm sequences. We then designed a nested PCR strategy to determine which of the putative splice variants of the remaining four genes were transcribed using cDNA prepared from seven life cycle stages of *S. mansoni* as well as isolated heads and tails of cercariae (Table 1). Nine of the variants were detected in every life-stage. TpmI.1 and TpmII.2 were largely restricted to the snail stages and TpmII.5 was only present in stages other than those in the snail. We were unable to detect the predicted TpmII variants 1, 10, 11 or 12 or variant 3 of Smp_085290. Overall, at least 12 isoforms from four Tpm genes were confirmed present in the *S. mansoni* transcriptome (Table 1).

### 3.2 TpmII

**Fig. 1:**
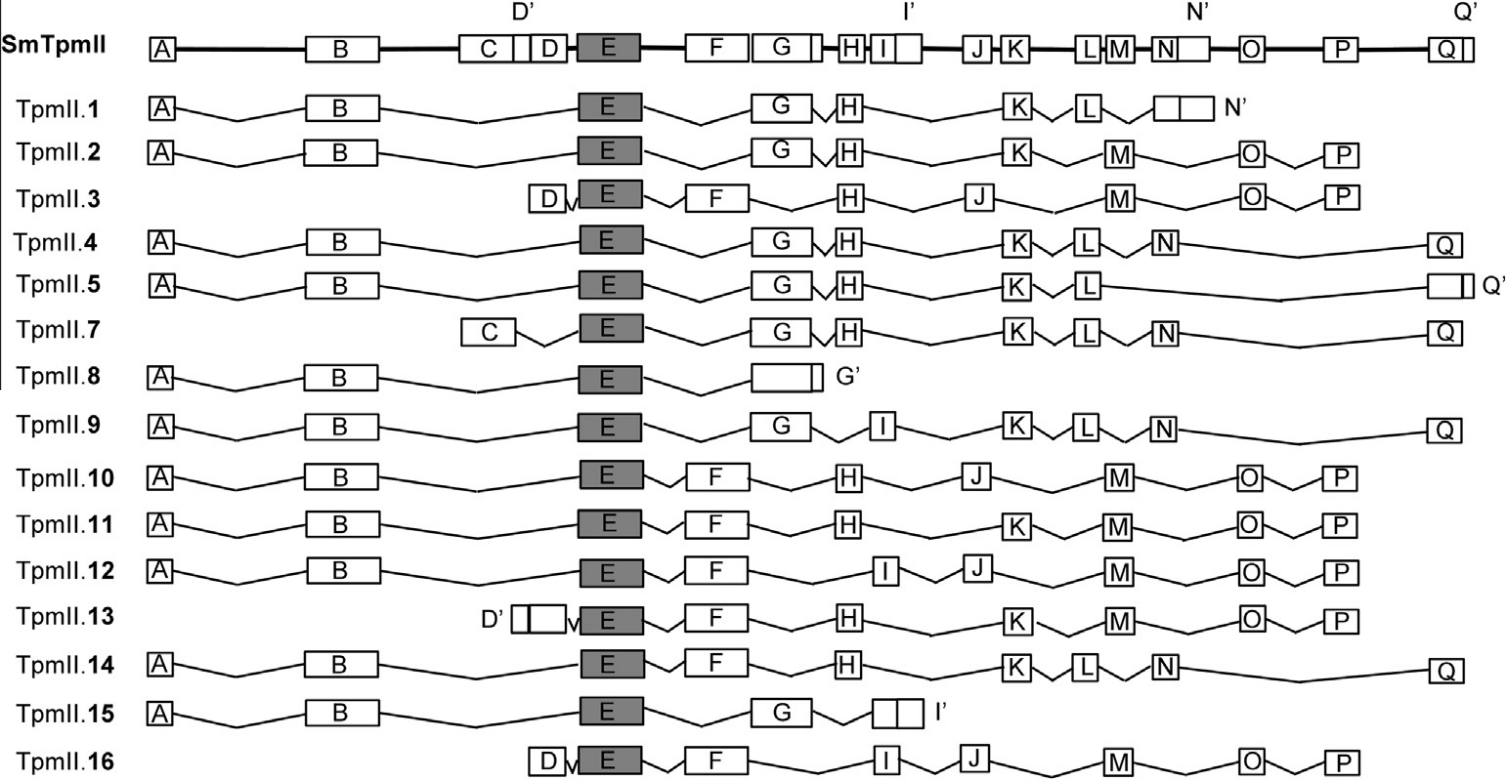
Exon map of *Schistosoma mansoni* tropomyosin (SmTpm)II. The annotated genome of *S. mansoni* predicts 17 exons for TpmII that are termed A–Q. Exon maps of the 14 genome-predicted splice variants and a further variant identified in the current study (TpmII.16) are shown. The common exon (E) is shaded grey. Some exons have additional sequences when they form the 5′ or 3′ end of the coding region (giving sequences D′, G′, N′ and Q′).

Since the majority of detectable Tpm isoforms arise from TpmII, we decided to focus our study on this gene (Smp_031770) and a detailed exon chart of *S. mansoni* TpmII is shown in Fig. 1. The tandem duplication of exons is apparent (exons F/G, H/I, J/K, L/M, N/O and P/Q) as is the fact that the duplicated exons, which typically share approximately 50% identity, are spliced in a mutually exclusive manner, i.e. no isoforms contain both ‘versions’ of any pair. Fig. 1 also includes TpmII.16, a splice variant (not predicted computationally) that was amplified and sequenced during the cloning of the coding region of TpmII.3 (see Section 2.3).

### 3.3 Quantitation of TpmII in different life stages

**Fig. 2:**
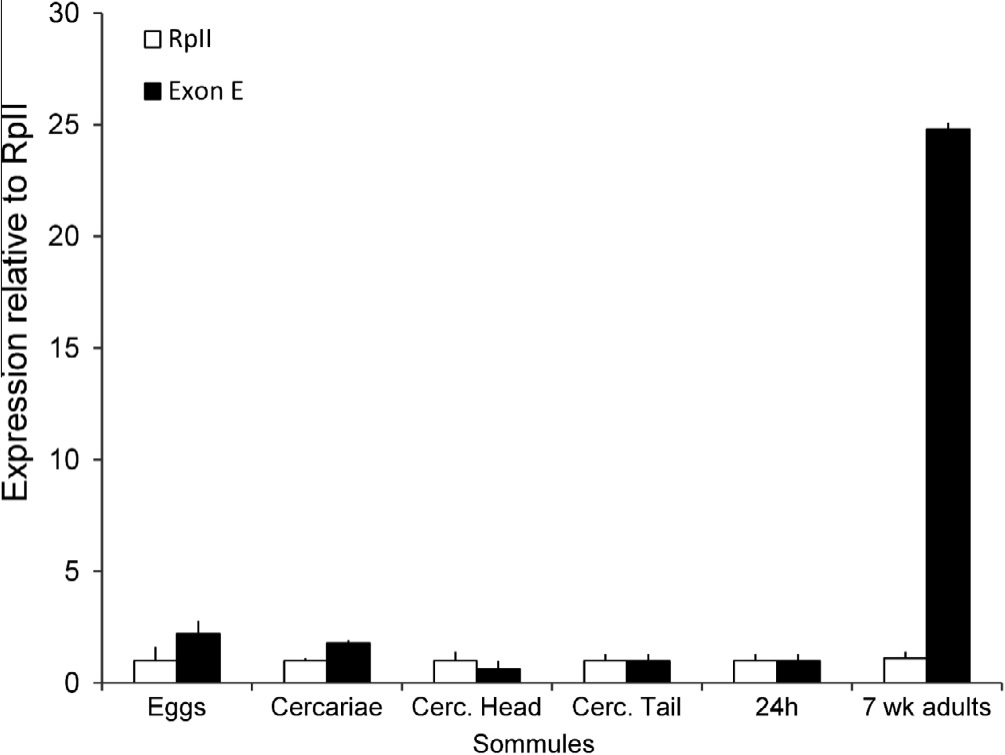
Quantitation of *Schistosoma mansoni* tropomyosin (Tpm)II transcription in different life stages. Quantitative real-time PCR was used to measure transcription levels of the common TpmII exon E, using cDNA prepared from the parasite material indicated (cerc., cercariae). Transcription of the stably expressed *S. mansoni* RNA polymerase II (Fitzpatrick et al., 2009; Liu et al., 2012), was measured simultaneously as a reference. Measurements were made in quadruplicate; shown are the mean ± S.E.M.

It was not possible to quantify individual splice variant transcripts by qPCR. Whilst non-quantitative differentiation was achieved using nested conventional PCR (Table 1), primers for qPCR cannot be nested and whilst some isoforms could be distinguished by amplifying entire coding regions in one step, the resulting amplicons (500–850 bp), were too large for qPCR methods. It was possible, however, to use qPCR on the common “E” exon to measure total TpmII transcription across the different life stages. Parasite material was chosen to be representative of life stages experienced by the human host; eggs, cercariae, 24 h schistosomula and 7 week adults. Fig. 2 shows that total transcription of TpmII was considerably higher in adult worms than in the other life stages.

### 3.4 Selecting splice variants for recombinant protein studies

**Fig. 3:**
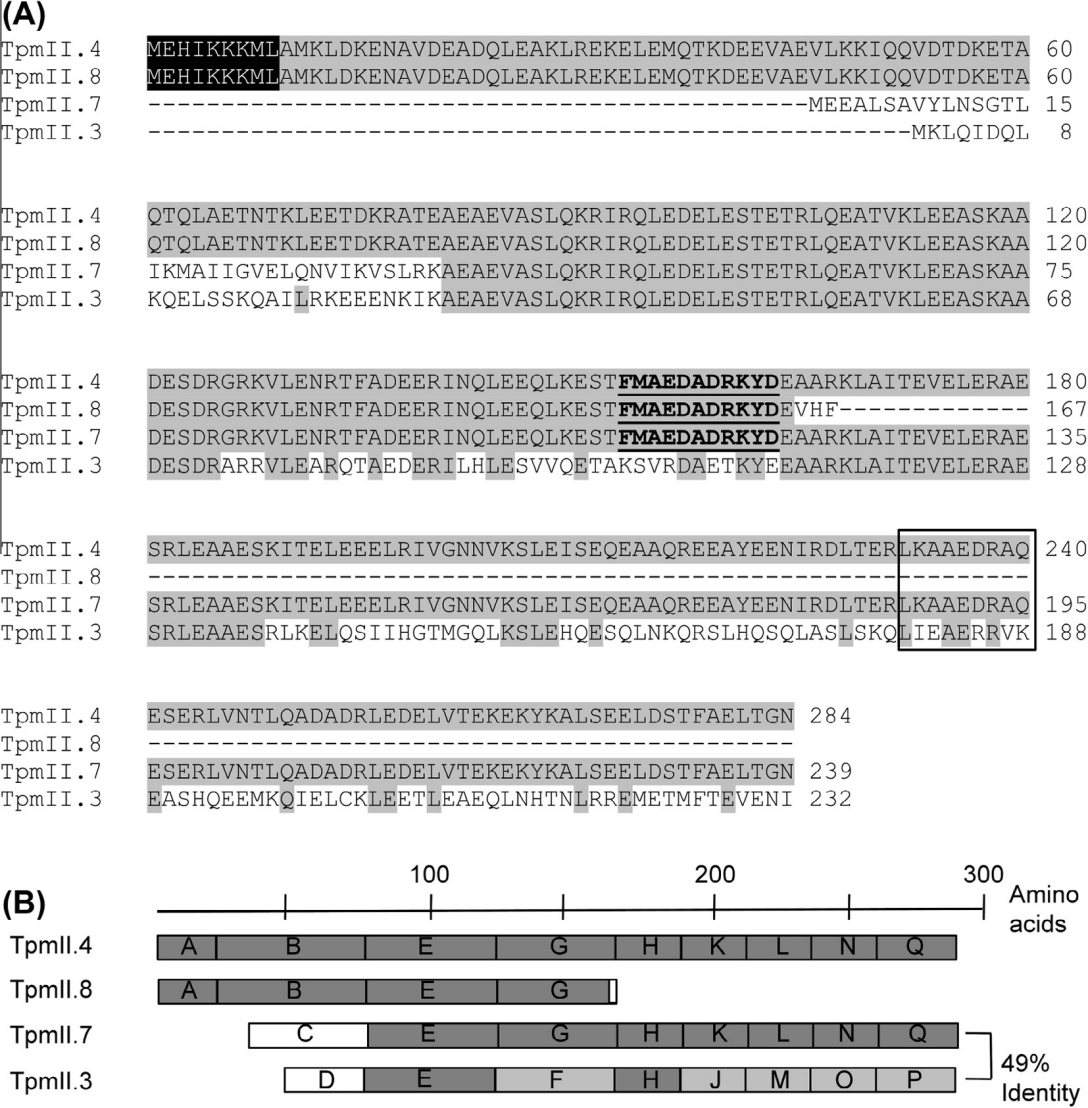
Alignment of the selected *Schistosoma mansoni* tropomyosin (Tpm) splice variants. The amino acid sequences of the four TpmII splice variants selected for cloning (Tpm II.4, 8, 3 and 7) are aligned (A) with the conserved N-terminal putative “muscle interaction” sequence MEHIKKKML (Smillie, 1979) highlighted in black and the sequences matching immunodominant B-cell epitope [F L]-L-A-E-[E D]-A-D-R-K-Y-D from *Onchocerca volvulus* (Jenkins et al., 1998) are underlined. The Tpm “signature” sequence L-K-[E A D]-A-E-x-R-A-[E T] is indicated by the box. Identical residues are in grey. (B) Protein structures generated by these sequences are illustrated with contributing exons and their degree of identity to TpmII.4 are indicated. Dark grey, 100%; light grey, 25–65%; and white, <10% identity.

After comparing predicted amino acid sequences of all the TpmII splice variants, four disparate examples (TpmII.3, 4, 7 and 8) were chosen in the hope of subsequently capturing a representative immunological profile of the entire repertoire of splice variants of this gene. Amino acid sequences of the four variants are aligned in Fig. 3A. The nine N-terminal residues of TpmII.4 and TpmII.8 (MEHIKKKML) are characteristic of muscle Tpm (Smillie, 1979). These residues are heavily conserved across species, as they interact with actin and troponin to regulate muscle contraction (Smillie, 1979). In contrast, non-muscle Tpms tend to be shorter with more variable N-terminal regions (Smillie, 1979; Côté, 1983). Together, this suggests that splice variants 4 and 8 are muscle isoforms, whilst 3 are 7 are non-muscle. Tpm 3, 4 and 7 each possess the L-K-[E A D]-A-E-x-R-A-[E T] sequence (Fig. 3A), a heavily conserved Tpm “signature” motif. This is present in both muscle and non-muscle Tpms, within a C-terminal region that is believed to enable the proper folding of the molecules into their characteristic coiled-coil conformation (Smillie, 1979). Finally, an immunodominant B-cell epitope [F L]-L-A-E-[E D]-A-D-R-K-Y-D has been reported in Tpm from the nematode *Onchocerca volvulus* (Jenkins et al., 1998) that coincides with an IgE binding epitope in shrimp Tpm (Subba Rao et al., 1998). This motif is present in all of the selected isoforms.

Fig. 3B illustrates the different protein structures generated from these four combinations of exons. TpmII.4 is a highly representative, 284 residue “high molecular weight” isoform, whilst TpmII.8 is a truncated version, equivalent to the N-terminal half of TpmII.4. In contrast, TpmII.7 is identical to the C-terminal 70% of TpmII.4, but has a unique non-homologous N-terminal domain. TpmII.3 is similar to TpmII.7 in size, but has its own unique N-terminal sequence and the high degree of exon substitution in other regions means that TpmII.3 shares less than 50% identity with TpmII.7 overall (see Fig. 3B).

### 3.5 Expression and characterisation of recombinant TpmII splice variants

**Fig. 4:**
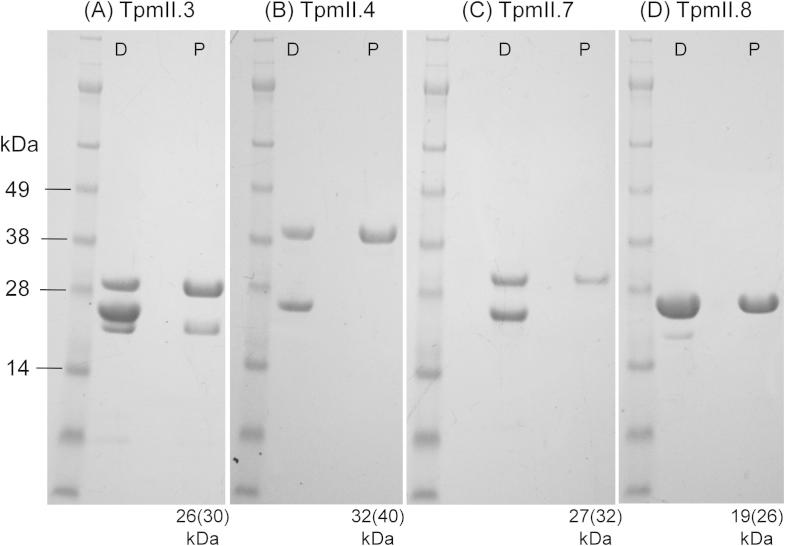
Purified recombinant *Schistosoma mansoni* tropomyosin (Tpm)II antigens. The four splice variants were expressed in *Escherichia coli* as GST fusion proteins, which were then cleaved with thrombin and purified using glutathione-agarose chromatography. Shown are the thrombin digests (D) and purified TpmII proteins (P) separated on SDS–PAGE. Tpm migrates anomalously in gel electrophoresis and the predicted and apparent (in parentheses) molecular weights are indicated .

The four selected splice variants were expressed in bacteria and Fig. 4 shows SDS–PAGE analysis of the recombinant proteins. As observed for Tpm in other species (Jenkins et al., 1998) all four migrated as greater than their predicted molecular weight values (Fig. 4). Additional bands were sometimes seen in preparations of TpmII.3 and TpmII.7 (e.g. Fig. 4). These were shown to be fragments of the respective Tpm isoforms by western blotting and N-terminal sequencing (not shown).

**Fig. 5:**
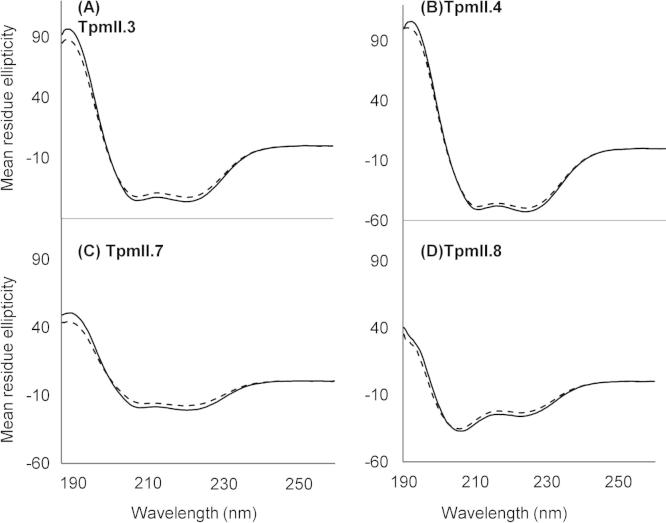
Circular dichroism (CD) spectra of helical *Schistosoma mansoni* tropomyosin (Tpm)II splice variants. The UV CD spectra of TpmII.3, 4, 7 and 8 are shown before thermal denaturation (solid lines) and after refolding by slow cooling (dashed lines).

Even though it is widely held that conformation plays an important role in immunological activity, recombinant antigens used for immunological testing are rarely tested to ensure proper folding. In this study conformation was assessed. First, TpmII isoforms were visualised using homology modelling to predict three-dimensional structures on the SwissModel webserver (http://swissmodel.expasy.org/). The same coiled-coil alpha-helical structure was obtained for each of the four proteins, typical of chain C crystal structure for Tpm (PDB ID: 1c1g.C, not shown). Then, to ensure that the secondary structure of the recombinant antigens was as predicted by the modelling, we performed CD spectroscopy on all four purified Tpm proteins. This indicated that the recombinant TpmII.3, 4 and 7 were indeed entirely alpha-helical, but that TpmII.8 contained a small, more disordered helical component at room temperature (Table 2). The thermal stability of the secondary structure of the molecules was investigated by heating from 25 to 95 °C, measuring CD at 222 nm (helix ‘signature’ wavelength) and calculating the melting temperatures (Table 2). When the proteins were cooled to 25 °C again, they refolded almost completely (Table 2). Wavelength scans of the antigens before and after thermal denaturation are shown in Fig. 5. These are typical of proteins with high alpha-helical content.

### 3.6 Anti-TpmII.3 IgE levels were associated with immunity to re-infection

**Fig. 6:**
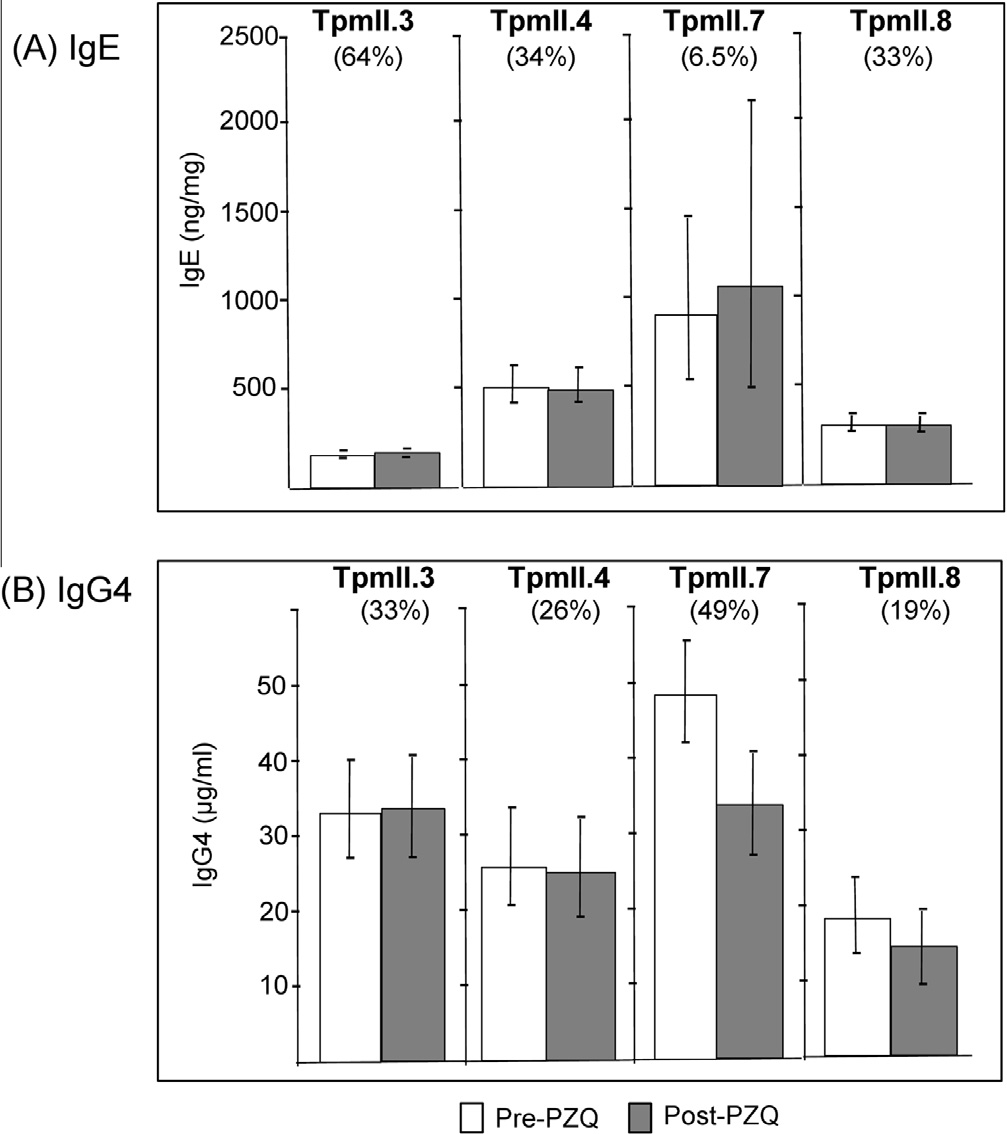
Levels of IgE and IgG4 to *Schistosoma mansoni* tropomyosin (Tpm)II isoforms. Levels of IgE (A) and IgG4 (B) to TpmII isoforms were measured in a population of infected boys and men (*n *= 228) from an area endemic for *S. mansoni*, both before and 7 weeks after treatment with Praziquantel. Shown are the levels (geometric mean ± 95% confidence interval) for those with a detectable response. The prevalence (%) of each response (before treatment) in the cohort is indicated.

The purified recombinant antigens were used to measure TpmII isoform-specific antibody responses in a cohort of 228 *S. mansoni*-infected males. Samples were obtained before and 9 weeks after PZQ treatment. For those that did produce a detectable response, levels of specific IgE and IgG4 are shown in Fig. 6A and B, respectively. Treatment did not affect levels of IgE or IgG4 to any of the four isoforms tested (Fig. 6, *P* > 0.1). With the exception of a post-treatment drop in the proportion of individuals with a detectable TpmII.7-IgG4 response (Tukey’s Honestly Significant Difference (HSD) test; *P* = 0.03), prevalence of responders was also not affected by treatment (*P* > 0.1). For each of the four Tpm isoforms, IgE and IgG4 responses were often present in the same individuals. That is, the two responses were not mutually exclusive and the less prevalent response was detected in a subset of those with the more prevalent response.

**Fig. 7:**
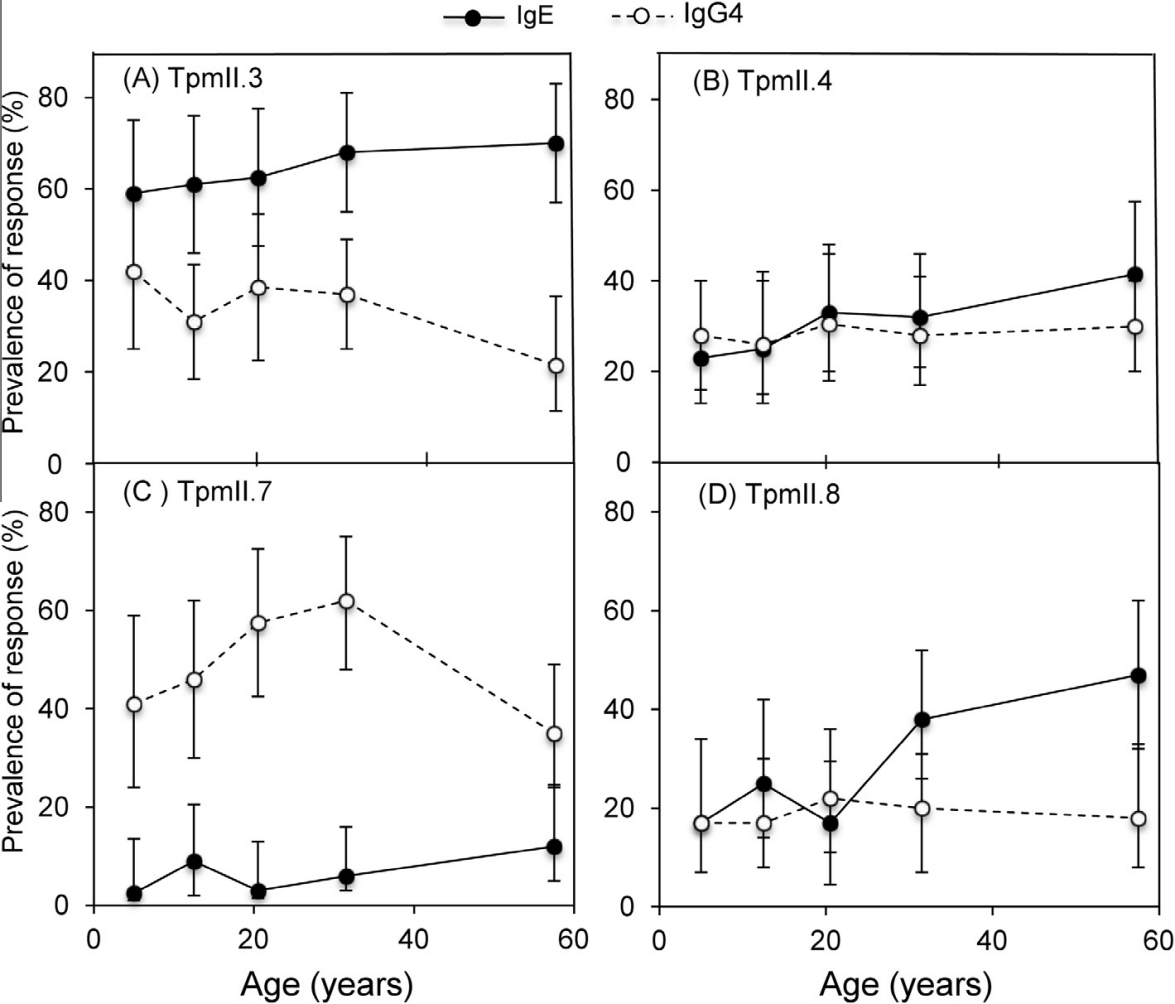
Prevalence of anti-*Schistosoma mansoni* tropomyosin (Tpm)II IgE and IgG4 responses by age of participant. The pre-treatment prevalence data for the IgE and IgG4 responses to (A) TpmII.3, (B) TpmII.4, (C) TpmII.7 and (D) TpmII.8 are shown for five age groups: 7–9 years (*n *= 39), 10–14 years (*n *= 46), 15–25 years (*n *= 45), 26–36 years (*n *= 50) and > 37 years (*n *= 48). The% of each group with a detectable response is plotted against the mean age of the group. Error bars represent 95% confidence intervals.

Fig. 7 displays prevalence of detectable anti-TpmII IgE and IgG4 responses over age. Interestingly, age does not have a strong effect on any of the anti-TpmII responses studied. There is a trend for TpmII.4–IgE and Tpm.8–IgE to increase with age but this does not reach statistical significance (Fig. 7B, D). The most prevalent IgE response was that to TpmII.3 (Fig. 7A); over 60% (*n *= 146) of the whole cohort had detectable IgE to TpmII.3, significantly more than to the other three isoforms (Tukey’s HSD test; *P *< 0.05). In contrast, IgE to TpmII.7 was very rare (6.5% had a detectable response, *n *= 14) and significantly less prevalent than IgE to the other three isoforms (*P *< 0.05). The most prevalent IgG4 response was that to TpmII.7 (Fig. 7C), detected in 49% of the cohort (*n *= 112), significantly more than IgG4 to TpmII.3, 4 or 8 (Tukey’s HSD test; *P* < 0.05).

Finally, linear regression analysis was performed to assess the relationship between post-treatment antibody levels against the four *S. mansoni* TpmII isoforms and immunity to re-infection, allowing for age. Anti-TpmII.3 IgE levels were significantly negatively correlated with re-infection intensity 2 years post-treatment (*β* = −0.64; *P* < 0.05, Table 3), whilst anti-TpmII.7 IgG4 levels were significantly positively correlated with re-infection intensity (*β* = 0.14; *P* < 0.05, Table 4). No other associations were observed (data not shown).

## 4 Discussion

Alternative splicing of mRNA is an important process in *Schistosoma* spp., contributing to the range of proteins required for parasite development and interaction with the host (Verjovski-Almeida and DeMarco, 2011). The current study demonstrates that the SmTpm genes are marked examples. In vertebrates, alternative splicing of Tpms is a well-characterised process generating many isoforms to carry out the protein’s different roles in muscle contraction and in the function and structures of the actin cytoskeleton (Perry, 2001). There is evidence that this is also the case with invertebrates and the current study confirms that in *S. mansoni*, four Tpm genes produce at least 13 splice variants.

In vertebrates, different isoforms of Tpm are expressed in different tissues and at different developmental stages (Perry, 2001). We did not see tissue variations in SmTpm isoform profiles when for example, comparing cercarial heads and tails, but there were some differences in developmental expression across the life cycle. For example, two of the isoforms were mutually exclusive. TpmII.2 was expressed only in the intermediate host and TpmII.5 only in the definitive host. Interestingly, these isoforms are almost identical except that different 3′ exons generate entirely non-homologous C-terminal domains (<15% identity). Most of the SmTpm isoforms are, however, transcribed in all life stages, so any differences in anti-isoform immune responses are unlikely to be attributed to developmental expression patterns in the way there have been with the SmTAL protein family (Fitzsimmons et al., 2007, 2012a).

Whilst unable to measure the mRNA amounts of different splice variants, we were able to show that overall expression of the TpmII gene was by far the greatest in the adult parasite. Evidence from vertebrate species would suggest that muscle and non-muscle isoforms have notable structural differences (Smillie, 1979; Côté, 1983). Muscle Tpm has a highly conserved N-terminal domain, whilst this is replaced in non-muscle isoforms, which tend to be shorter overall. Mature schistosomes, especially the males, are muscular creatures and muscle Tpm may be abundant. However immunohistology studies on adult parasites (MacGregor and Shore, 1990) indicate that most Tpm is non-muscle, found in other parenchymal cells and in the tegument – the outer layer that covers the muscle and acts as an interface with the host.

Some of the splice variants are very similar to each other (e.g. TpmII.2 and TpmII.5 above). The swapping of a small number of exons may make a functional difference to the protein but not greatly influence antigenicity. For immunological study however, we chose four isoforms, TpmII.3, 4, 7 and 8, with marked regional differences, especially in their N and C-terminal domains. TpmII.4 is a highly representative 284 residue, muscle-type isoform. It is homologous to known Tpm allergens from shrimp (Pen a1) and other invertebrates (Ayuso et al., 2002; Sereda et al., 2008), including five sections of these molecules proposed to contain continuous IgE epitopes (Sereda et al., 2008). Whilst SmTpmII.4 contains sequences homologous to all five sections (Sereda et al., 2008), TpmII.8 is truncated before the two C-terminal epitopes, and TpmII.3 and TpmII.7 have entirely different N-terminal sections. Moreover the unique N-terminal domains of TpmII.3 and TpmII.7 are likely to contain other epitopes specific for those isoforms.

Despite all the published data on continuous epitopes, it seems that, in the major allergens at least, most IgE epitopes are of the conformational (discontinuous) type (Arnon and Van Regenmortel, 1992; Valenta et al., 2004). Correct folding of antigens is therefore crucially important and CD confirmed that all the tested Tpm isoforms possessed the expected coiled-coil secondary and tertiary structure. The native Tpm protein is a dimer of two Tpm chains, which can associate end to end to form larger filamentous multimers (Perry, 2001). Therefore it is possible that the quaternary structure may generate further conformational epitopes. It is not known whether these phenomena occur with the recombinant SmTpm, but gel exclusion chromatography indicated that at least one isoform, SmTpmII.4, formed large multimers in solution (unpublished data).

Only a small number of protein families contain allergens/IgE-antigens (Radauer, 2008) and Tpms represent some of the most common (Reese et al., 1999). We believe this is the first report of an IgE response to schistosome Tpm. In this study IgE from infected individuals bound to all four TpmII isotypes tested. Many individuals also produced IgG4 that bound the four proteins. IgG4 is regarded as a blocking antibody, competing with IgE for antigen binding and acting through inhibitory FcgRIIB to suppress FceRI-mediated activation of IgE-effector cells (Kepley et al., 2000; Smith and Clatworthy, 2010). Whilst IgE has been associated with resistance to re-infection, specific IgG4 responses, or rather high IgG4:IgE ratios, have been correlated with susceptibility (Hagan et al., 1991; Demeure et al., 1993; Li et al., 2001; Jiz et al., 2009; Pinot de Moira et al., 2010).

There were significant differences between the antibody responses to the four different isoforms. Most striking was the difference between the two non-muscle molecules, TpmII.3 and TpmII.7. The response to TpmII.3 was predominantly IgE, with few people producing TpmII.3–IgG4. The response to TpmII.7 was predominantly IgG4 with few individuals producing IgE. Given previous work linking the IgE:IgG4 balance to immunity, it is notable that those with the IgE-dominated response to TmpII.3 were more resistant to re-infection and those with the IgG4-dominated response to TpmII.7 were more susceptible. The antibody responses to the muscle-type TpmII.4 and its truncated form, TpmII.8, were very similar. They share many of the same epitopes, have the same developmental expression pattern and if they are both in the muscle, are likely to have a similar exposure pattern to the host immune system. We have not established why the two non-muscle forms evoke such different responses, although they do have entirely different N-terminal domains and the rest of the molecule is also relatively dissimilar. Nothing is known about their tissue distribution or their accessibility to the host immune system but these may be important factors in their antigenicity.

Loverde and colleagues showed that individuals infected with *S. mansoni* produced IgG against parasite Tpm (Xu et al., 1991) but ours is the first known report correlating the anti-Tpm response with immunity. Invertebrate Tpm is a major clinical allergen, shown to be targeted by human IgE in a number of helminth infections, now including schistosomes. Alternative splicing is an important component of Tpm biology and this work shows it is a key consideration in studying the IgE response to this allergenic molecule.

## Figures and Tables

**Table 1:** Detection of *Schistosoma mansoni* tropomyosin transcripts throughout the life-cycle using conventional nested PCR.

Tropomyosin^a^	Eggs^b^	Mir.	24 h Spor.	99 h Spor.	Cerc.	Cerc. tails	Cerc . heads	24 h Som.	7 wk Adult
TpmI.1	+	+	+	+				+	
TpmI.2	+	+	+	+	+	+	+	+	+
TpmII.1									
TpmII.2		+	+	+					
TpmII.3	+	+	+	+	+	+	+	+	+
TpmII.4	+	+	+	+	+	+	+	+	+
TpmII.5	+				+	+	+	+	+
TpmII.7	+	+	+	+	+	+	+	+	+
TpmII.8	+	+	+	+	+	+	+	+	+
TpmII.9	+	+	+	+	+	+	+	+	+
TpmII.10									
TpmII.11									
TpmII.12									
TpmII.13	+	+	+	+	+	+	+	+	+
TpmII.14									
TpmII.15									
Smp_022170.1	+	+	+	+	+	+	+	+	+
Smp_085290.1^c^	+	+	+	+	+	+	+	+	+
Smp_085290.3									

a All genes and splice variants annotated as “tropomyosin” in the *S. mansoni* genome.

b Template cDNA was prepared from eggs, miracidia (Mir.), 24 h sporocysts (Spor.), 99 h sporocysts, whole cercariae (Cerc.), cercarial tails, cercerial heads, 24 h schistosomula (Som.) and 7 week (wk) mixed-sex adults.

c Isoforms of Smp_085290.1 and Smp_085290.2 differ only in one codon and were not considered separately.

**Table 2:** Circular dichroism analysis of purified *Schistosoma mansoni* tropomyosin (TpmII) proteins.

TpmII isoforms	Tm^a^ (°C)	% Alpha-helix^b^ (before heating)	% Alpha-helix (after heating)
TpmII.3	52	100	100
TpmII.4	40	100	100
TpmII.7	38	100	90.9
TpmII.8	30	95.4	91.5

a Melting temperatures (Tm) were calculated by taking the maxima of the derivatives of thermal denaturation curves. Normalised root-mean-squared S.D. terms for all secondary structure determinations were <0.1.

b Percent helicity calculations were performed using the CONTINLL analysis program and the SP175 190–240 nm reference data set on the Dichroweb interface.

**Table 3:** Age-adjusted linear regression model of participant re-infection with *Schistosoma mansoni* 2 years post-treatment.

Parameter		*β* (S.E.)		*P*
Intercept		7.45	(1.631)	<0.001
Age (versus 7–9 year age group)	10–14 yrs	0.034	(0.514)	<0.001
	15–25 yrs	−0.747	(0.548)	
	26–36 yrs	−2.073	(0.51)	
	37–76 yrs	−2.548	(0.52)	
Anti-SmTpmII.3 IgE levels		−0.641	(0.326)	0.05

SmTpm, *Schistosoma mansoni* tropomyosin.

**Table 4:** Age-adjusted linear regression model of participant re-infection with *Schistosoma mansoni* 2 years post-treatment.

Parameter		*β* (S.E.)		*P*
Intercept		4.626	(0.385)	<0.001
Age (vs. 7–9 years)	10–14 yrs	−0.021	(0.508)	<0.001
	15–25 yrs	−1.01	(0.549)	
	26–36 yrs	−2.403	(0.498)	
	37–76 yrs	−2.49	(0.501)	
Anti-SmTpmII.7 IgG4 levels		0.138	(0.049)	0.006

SmTpm, *Schistosoma mansoni* tropomyosin.
