# Supplementary material for: Human IgE responses to different splice variants of Schistosoma mansoni tropomyosin: associations with immunity
Source: Int J Parasitol. 2014 May;44(6):381–90. doi: 10.1016/j.ijpara.2014.02.004 (PMC4026961; doi:10.1016/j.ijpara.2014.02.004)
Supplement: Supplementary data [file mmc1.docx]

**Supplementary Table S1.** Primers used to detect *Schistosoma mansoni* tropomyosin isoforms.

| **Tropomyosin Isoforms** | **(+) Detection Primer** | **(-) Detection Primer** |
| --- | --- | --- |
| Smp_022170.1 (703 bp) | 5’-GTC AGT ATG ATA AGT GCT AAA C-3’ | 5’-CCA TGT TAA TTT GTT CTT GAA C-3’ |
| Smp_031770.1 (780 bp) | 5’-AAG AGA ATG CTG TAG ATG AAG C-3’ | 5’-ATG TAG GGG TAA TCG TAT GC-3’ |
| Smp_031770.2 (322 bp) | 5’-TCT ACA TTT ATG GCT GAA-3’ | 5’-CTT CTA ATT TAC ACA ATT CG-3’ |
| Smp_031770.3 (420 bp) | 5’-GAA AAT AAA ATC AAG GCC GAA GCT-3’ | 5’-ATT TAA CTG AGA TTC TTG GTG CTC-3’ |
| Smp_031770.4 (565 bp) | 5’-CAG ATA AAC GAG CAA CTG AGG-3’ | 5’-GTG ACC AAT TCA TCT TCA AGA CGA-3’ |
| Smp_031770.5 (671 bp) | 5’-AAG AGA ATG CTG TAG ATG AAG C-3’ | 5’-TGA CCA ATT CAT AGC TTT TAA GC-3’ |
| Smp_031770.7 (555 bp) | 5’-CTT ACG AAA GGC CGA AGC TGA-3’ | 5’-GTG ACC AAT TCA TCT TCA AGA CGA-3’ |
| Smp_031770.8 (461 bp) | 5’-AAG AGA ATG CTG TAG ATG AAG C-3’ | 5’-TTA GAA ATG CAC CTC ATC GTA-3’ |
| Smp_031770.9 (322 bp) | 5’-ACT CGA AAA CTT GCC GTT GC-3’ | 5’-GAA TCC AAT TCT TCA CTG AGT GC-3’ |
| Smp_031770.10 (535 bp) | 5’-AAG AGA ATG CTG TAG ATG AAG C-3’ | 5’-AGC TCC TTT AAA CGA CTT TC-3’ |
| Smp_031770.11 (609 bp) | 5’-AAG AGA ATG CTG TAG ATG AAG C-3’ | 5’-GTT GTT TAT TTA ACT CTT GTT CAG A-3’ |
| Smp_031770.12 (535 bp) | 5’-AAG AGA ATG CTG TAG ATG AAG C-3’ | 5’-AGC TCC TTT AAA CGA CTT TC-3’ |
| Smp_031770.13 (422 bp) | 5’-TAA AAT CAA GGC CGA AGC T-3’ | 5’-GTT GTT TAT TTA ACT CTT GTT CAG A-3’ |
| Smp_031770.14 (350 bp) | 5’-TCT GTG GTA CAA GAA ACT GC-3’ | 5’-GTG ACC AAT TCA TCT TCA AGA CGA-3’ |
| Smp_031770.15 (535 bp) | 5’-AAG AGA ATG CTG TAG ATG AAG C-3’ | 5’-TCT AAG AAG AAG TAC CTT TCT GC-3’ |
| Smp_044010.1 (716 bp) | 5’-GAC TGT CGT CAA GAG GAA A-3’ | 5’-TAA GTT CTG CGA AAG TTT GAT-3’ |
| Smp_044010.2 (812 bp) | 5’-GAA ATT AGA GAA AGA GAA TGC TAT -3’ | 5’-TAA GTT CTG CGA AAG TTT GAT-3’ |
| Smp_085290.1/2 (615 bp) | 5’-TT CTG AGG AAG CGA CTG AAT-3’ | 5’-GAT CGA GTG TAG CAA TGT TTC-3’ |
| Smp_085290.3 (584 bp) | 5’-TT CTG AGG AAG CGA CTG AAT-3’ | 5’-CTA TAA TCT TAA ATA AAC CCT CTG C-3’ |

**Supplementary Table S2.** Nested primers used to distinguish closely-related *Schistosoma mansoni* tropomyosin isoforms.

| **Tropomyosin Isoforms** | **(+) Nesting Primer** | **(-) Nesting Primer** |
| --- | --- | --- |
| Smp_031770.4a (to rule out isoform 9) (276 bp) | 5’-TAT CAC GGA AGT GGA ACT CGA ACG-3’ | 5’-GTG ACC AAT TCA TCT TCA AGA CGA-3’ |
| Smp_031770.4b (to rule out isoform 14) (*NOT* 350 bp) | 5’-TCT GTG GTA CAA GAA ACT GC-3’ | 5’-GTG ACC AAT TCA TCT TCA AGA CGA-3’ |
| Smp_031770.10 (498 bp) | 5’-AAG AGA ATG CTG TAG ATG AAG C-3’ | 5’-ACT CAG CCC GTT CGA GTT-3’ |
| Smp_031770.11 (241 bp) | 5’-GAC GAG CGG ATT TTA CAT TTG-3’ | 5’-GTT GTT TAT TTA ACT CTT GTT CAG A-3’ |
| Smp_031770.12 (497 bp) | 5’-AAG AGA ATG CTG TAG ATG AAG C-3’ | 5’-TTC AGC GTG AGA TAG TGC G-3’ |

**Supplementary Table S3.** Primers with appended restriction sites (in bold) for cloning *Schistosoma mansoni* tropomyosin II variant (SmTpmII) sequences into expression vector pGEX-KG.

| **Tropomyosin Variant** | **(+) pGEX Cloning Primer** | **(-) pGEX Cloning Primer** |
| --- | --- | --- |
| SmTpmII.3 (699 bp) | 5’-AA **GGA TCC** ATG AAG CTT CAG ATA GAC-3’ | 5’-GGC **TCT AGA** TTA GAT ATT TTC TAC TTC AGT-3’ |
| SmTpmII.4 (855 bp) | 5’-AA **GGA TCC** ATG GAA CAT ATT AAA AAG-3’ | 5’-GGC **TCT AGA** TTA GTT TCC AGT AAG TTC TG-3’ |
| SmTpmII.7 (720 bp) | 5’-GC **TCT AGA** C ATG GAA GAA GCT TTA TCA-3’ | 5’-GC **CTC GAG** TTA GTT TCC AGT AAG TTC TG-3’ |
| SmTpmII.8 (504 bp) | 5’-AA **GGA TCC** ATG GAA CAT ATT AAA AAG-3’ | 5’-AA **TCT AGA** TTA GAA ATG CAC CTC ATC-3’ |
